# Successful application of focused extracorporeal shockwave therapy for plantar fasciitis in patients suffering from metastatic breast cancer

**DOI:** 10.1007/s00520-021-06117-9

**Published:** 2021-03-21

**Authors:** Andrej Zdravkovic, Michael Mickel, Richard Crevenna

**Affiliations:** Department of Physical Medicine, Rehabilitation and Occupational Medicine, Medical University of Vienna, Waehringer Guertel 18-20, A-1090 Vienna, Austria

**Keywords:** Extracorporeal shockwave therapy, Plantar fasciitis, Breast neoplasms, Metastatic bone disease

## Abstract

**Purpose:**

Focused extracorporeal shock wave therapy (fESWT) has been shown to be effective in a large number of musculoskeletal disorders. Until 2016, cancer was considered a contraindication for fESWT. The goal of this Commentary is to address the subject of fESWT in cancer patients and present a case of a successful application of fESWT in a breast cancer patient with metastatic bone disease, suffering from debilitating heel pain caused by plantar fasciitis.

**Methods:**

The subject of fESWT application in cancer patients is discussed using the example of a 75-year-old female with breast cancer and metastatic bone disease suffering from bilateral inferior heel pain, who was referred to our clinic with a tentative diagnosis of polyneuropathy. Patient history, clinical examination, electrodiagnostic testing, and radiological findings all indicated plantar fasciitis, rather than polyneuropathy. The possibility of metastatic bone lesions in the treatment area was excluded and the patient was thereupon treated with 5 weekly applications of low-energy fESWT.

**Results:**

The treatment lead to a reduction in pain of approximately 80% with no adverse events.

**Conclusion:**

fESWT may be a viable treatment option for plantar fasciitis even in cancer patients, provided certain conditions are met.

## Introduction

Extracorporeal shock wave therapy (ESWT) is a physical therapy modality in which pressure waves are generated and transmitted to body tissues [1]. Of the two existing types, namely focused and radial ESWT, only focused ESWT (fESWT) produces pressure waves, which have the typical characteristics of shockwaves [2]. The proposed mechanisms of action of ESWT include pain relief, possibly by means of hyperstimulation analgesia and the stimulation of tissue regeneration, in part due to increased matrix turnover and collagen production [1].

ESWT is successfully used to treat plantar fasciitis (PF) [3]. Although adverse effects are rare [4], ESWT is often neglected as a treatment option in cancer patients, as cancer was widely considered a contraindication for ESWT. In 2016, the International Society for Medical Shockwave Treatment issued a consensus statement on ESWT indications and contraindications, where tumors in the treatment area were classified as a contraindication, but not cancer per se as an underlying disease [5].

In this case study, the successful treatment of PF in a patient suffering from breast cancer with metastatic bone disease is described.

## Case presentation

### Patient history

A 75-year-old Caucasian female was referred to the Department of Physical Medicine, Rehabilitation and Occupational Medicine of the Medical University of Vienna with a diagnosis of polyneuropathy. The patient described a bilateral heel pain with a gradual onset, beginning approximately 4 months before the referral. The pain was of an undulating intensity, with maxima reaching 81 mm on a visual analog scale while walking upon getting out of bed. As the painful area was located medially on the heel, the patient was forced to put her weight on the lateral rim of the foot while walking.

The walking ability was severely reduced, with a maximum uninterrupted walking distance of approximately 100 m. The limiting factor was a pain in the lateral compartment of the left knee, which had become apparent about the same time as the heel pain. However, recurring knee pain had been known for several years before the referral.

She was suffering from breast cancer, which was diagnosed in 2014 and had been treated with surgery, followed by adjuvant chemotherapy. Metastatic bone disease was ascertained at the time of diagnosis and consisted of lesions in the spine, pelvic bones, and several ribs. No recent progression of the underlying disease had been observed.

### Clinical presentation

In the clinical examination, the heel pain was localized on both sides along the medial part of the insertion of the plantar aponeurosis on the calcaneus, as well as along the most medial bundle of its central part. Pressure on these areas elicited pain of the same quality and location as experienced during walking.

A recent bone scintigraphy showed no tracer uptake in the feet. Uptake in the medial condyle of the left femur and of the left tibia was consistent with osteoarthritis.

**Fig. 1:**
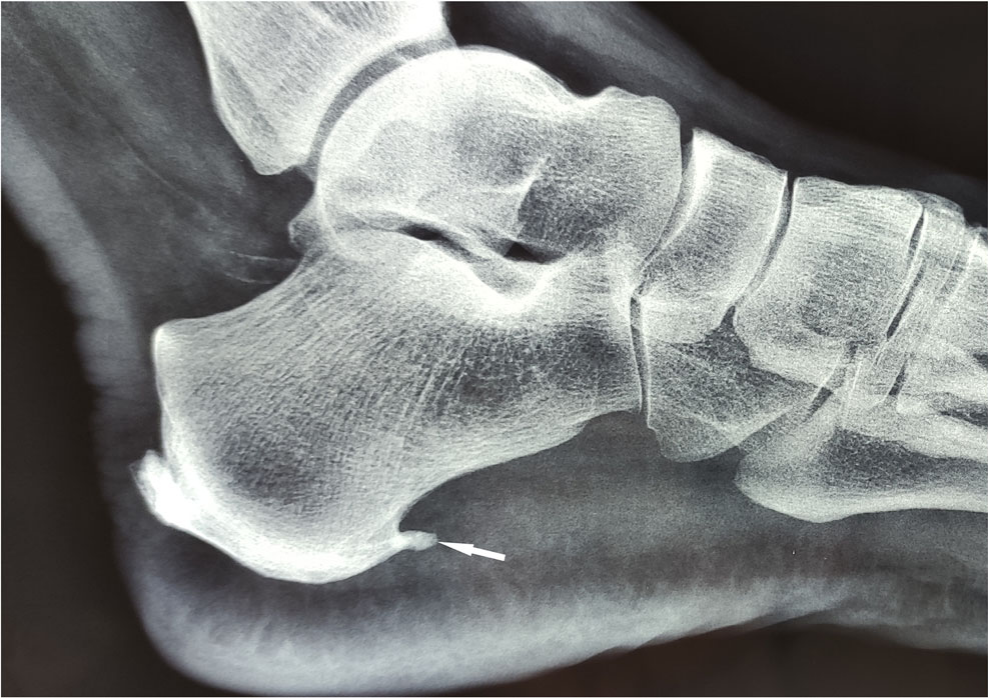
Plain radiograph of the left calcaneus. Arrow indicates plantar heel spur

Plain radiographs revealed a plantar heel spur on both sides, without indication of bone lesions. The plain radiograph of the left calcaneus is shown in Fig. 1.

Ultrasound showed a thickening of the plantar aponeurosis near the insertion on the calcaneus, with a thickness of 4.9 mm on the left side and 4.8 mm on the right side.

Polyneuropathy was excluded through electrodiagnostic testing.

### Intervention

fESWT was administered on both heels over 5 weekly sessions using the F10G4 therapy source of a PiezoWave 2 device (Richard Wolf GmbH, Knittlingen, Germany). One thousand five hundred impulses were administered on each foot with a frequency of 5 Hz, at a focus depth of 15 mm and an average energy flow density of approximately 0.39 mJ/mm^2^. The focus depth was chosen according to the thickness of the tissues superficial to the aponeurosis, measured during ultrasound imaging.

fESWT did not cause any adverse events. The reported pain intensity decreased gradually after each session. At week 6, the patient reported a maximum pain intensity of 14 mm on the visual analog scale. The patient reported no pain in the heels up to 12 weeks after fESWT, during which time she received physical therapy for knee pain due to osteoarthritis.

## Discussion

The patient was referred to our clinic with a tentative diagnosis of polyneuropathy and a substantial disease burden caused by bilateral heel pain. The initial diagnosis of polyneuropathy was not in line with the results of electrodiagnostic testing, and clinical and radiological findings. Although polyneuropathy cannot be considered a “zebra” in a cancer patient, there are usually multiple “horses” to be considered. Indeed, PF is the most common cause of inferior heel pain [6]. However, metastatic disease of the foot, though exceedingly rare, should be excluded in patients with malignant disease and foot pain [7].

In older adults, PF has been associated with a greatly increased prevalence of falls [8]. In addition, cancer patients seem to be at an increased risk of falling, and prior falls are associated with a worse outcome of cancer therapy [9, 10].

ESWT is an effective treatment option for PF [3] and the presence of a heel spur in lateral radiographs may present a positive prognostic factor concerning the success of ESWT [11]. Historically, cancer per se was considered a contraindication for ESWT, and this position changed only recently, following a consensus statement issued by the German Speaking International Society for Extracorporeal Shockwave Treatment [12], as well as the International Society for Medical Shockwave Treatment [5]. As exercise represents an important adjunct therapy with a substantial beneficial impact in cancer management [13], it stands to reason that an effective treatment of painful musculoskeletal disorders is of great importance in relation to the implementation of exercise guidelines.

Functional limitations, which in turn reduce the health-related quality of life, are highly prevalent in adults living with cancer, and a reduced walking ability may affect more than half of this patient group [14]. These limitations should therefore be treated with a specific, targeted treatment plan, in order to restore or at least improve the physical, and thusly psychosocial functioning of patients. At our hospital, an interdisciplinary, multimodal approach is the standard in terms of cancer rehabilitation and supportive care [15].

Osteoporosis, a condition for which breast cancer survivors are at an increased risk [16], is not considered a contraindication for fESWT [5]. Indeed, initial findings suggest a positive effect of fESWT on bone formation [17].

In our experience, cancer patients can profit from a multimodal approach to side-effect management and supportive care, and ESWT plays an important role in the treatment of a number of disorders, including PF [18–20].

## Conclusion

fESWT seems to be a safe, efficient, and cost-effective treatment option in cancer patients with PF and can be administered in an outpatient setting, provided that no metastatic lesions are present in the treatment area.

## Data Availability

All background information concerning the methodology of the intervention and creation of this paper is open for journal review if requested.
